# Rapid Fabrication of High-Aspect-Ratio Platinum Microprobes by Electrochemical Discharge Etching

**DOI:** 10.3390/ma9040233

**Published:** 2016-03-25

**Authors:** Min Zhang, Xiangwei Lian

**Affiliations:** Graduate School at Shenzhen, Tsinghua University, Shenzhen 518055, China; lianxw13@mails.tsinghua.edu.cn

**Keywords:** electrochemical discharge etching, microprobes, aspect ratio

## Abstract

Using a graphite crucible as the counter-electrode, platinum microprobes with an aspect ratio of 30 and a tip apex radius less than 100 nm were fabricated by an electrochemical discharge etching process. The “neck-in” structure on the platinum wire induced by the electrical discharge at the liquid-air interface plays a key role in the probe shape and the voltage of the following pure electrochemical etching determines the final probe aspect ratio and tip dimensions. Moreover, the shape and diameter of the graphite counter-electrode also exhibit a significant effect on the realization of high-aspect-ratio probes. The method presented here provides a simple and rapid approach to the fabrication of micro-tools for micromachining, micromanipulation, as well as biomedical applications.

## 1. Introduction

In recent years, micro/nanoprobes have been widely investigated for their application in scanning probe microscopy, micro/nanomanipulation, multipoint contact measurements, neural implants and electrochemical micromachining [1,2,3,4,5]. The tip size and shape of the probes play important roles in various applications [6,7]. Different techniques can be used to fabricate sharp tips, including cutting [8], mechanical pulling [9], grinding [10], ion milling [11], ion beam–induced deposition [12], electrochemical etching [13,14,15], and electrochemical machining [5]. Among these methods, electrochemical etching is the most widely explored technique for microprobe fabrication because of its low cost, reproducibility, and ease of implementation [16]. Tungsten is the most commonly used probe material when using electrochemical etching largely due to its high strength and the simple fabrication process. Tungsten probes with an ultra-high aspect ratio of 200 were fabricated by pulse electrochemical machining [5].

However, a tungsten tip obtained through a typical electrochemical etching process can easily be contaminated and oxidized [17]. This limits their application in situations where conductivity and chemical stability play important roles, including electrochemical micromachining and biomedical application. In this case, platinum is a better choice than tungsten because of its inertness and stability in various environments. The fabrication of platinum microprobes has been studied by a number of researchers, who have concluded that sharp tips with a low aspect ratio can be obtained easily [18,19]. However, few studies have been conducted on platinum probes with high aspect ratios.

We have fabricated platinum probes with aspect ratios from 10 to 30 and a tip apex radius less than 300 nm using a two-step electrochemical etching process [20]. In this paper, the etching process was further investigated and the voltage applied at the second etching step was refined. Probes with a tip radius less than 100 nm were obtained by appropriately selecting the diameter of the graphite crucible and carefully controlling the temperature, vibration and etching rate. Moreover, the effect of the shape and size of graphite electrodes on the profile and yield rate of platinum probes was also investigated.

## 2. Results and Discussion

**Figure 1:**
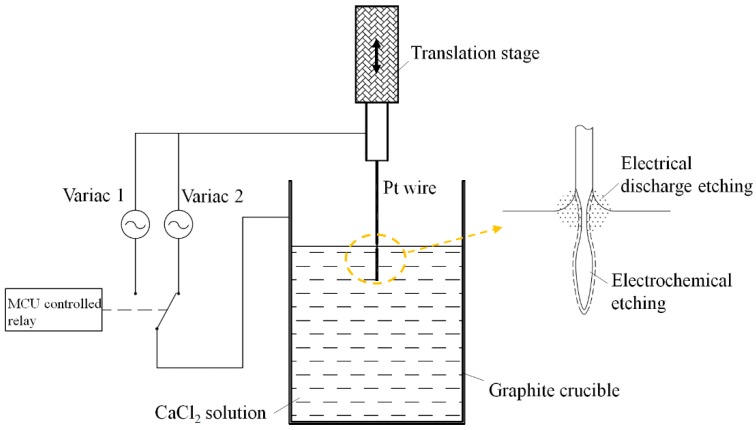
The schematic of the two-step etching process.

The full etching process is shown in Figure 1. In the first etching step, the platinum wire is immersed into the solution, pointing downwards about 3 mm. An AC voltage of 35 V is applied to the platinum wire and the counter-electrode. After 90 s, the voltage is then reduced to the second stage rapidly by a microcontroller unit (MCU) -controlled relay. The etching reaction will stop automatically when all of the platinum wire under the solution is etched out.

**Figure 2:**
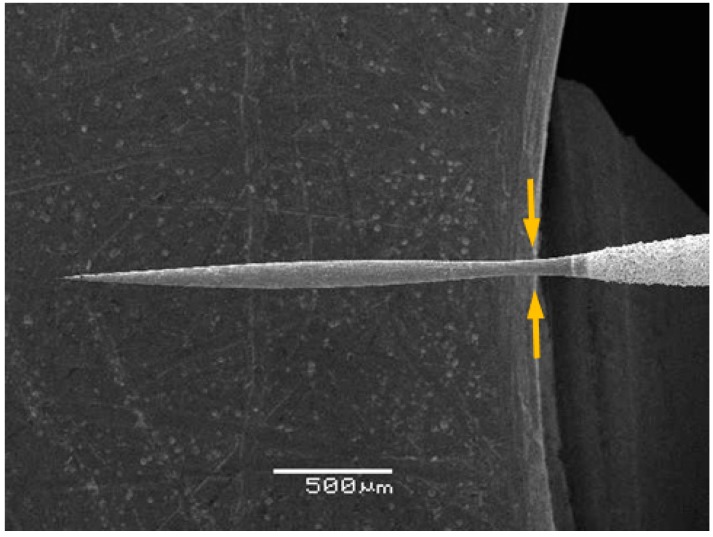
SEM image of the “neck-in” structure fabricated in the first etching step.

The initial voltage applied between the platinum wire and the graphite counter-electrode results in constant electric sparks at the specimen-liquid-air interface. In the meantime, the portion of the platinum wire immersed in the solution is shortened rapidly by the electrochemical reaction of platinum. This indicates that the etching process in the first step is a combination of electrochemical etching from the bottom and electrical discharge etching at the liquid surface, as shown in the inset of Figure 1. The electrochemical discharge etching at the liquid surface results in a much higher etching rate than the electrochemical etching inside the solution and, therefore, a “neck-in” shape of the platinum tip after the first-step etching. Figure 2 shows the SEM image of the platinum tip fabricated in the first etching step. The “neck-in” structure formed in the immediate vicinity of the liquid-air interface.

The electrochemical discharge etching process shifted at the second step to pure electrochemical etching with lower voltages. When the voltage is reduced from 35 to 24 V or below, the sparks on the air-liquid interface disappear. A slow and gentle reaction can help to improve the surface roughness caused by the violent electrical discharge etching in the first step, and moreover, to control the shape and sharpness of the final probe tip.

**Figure 3:**
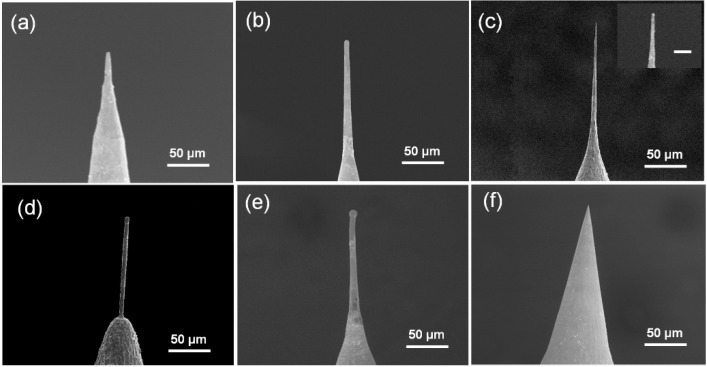
(**a**)–(**e**) SEM images of the as-fabricated Pt probes under different voltages of the second etching step. (**a**) 16 V; (**b**) 18 V; (**c**) 20 V; (**d**) 22 V; (**e**) 24 V. The inset in (**c**) shows the details of the probe tip. The scale bar is 1 μm. The diameter of the graphite crucible used is 30 mm; (**f**) Pt probe fabricated without electrical discharge etching.

Experiment results indicate that the probe fabrication process is quite sensitive to the second step voltage. A voltage lower than 18 V leads to a longer etching time and, therefore, the disappearance of the long neck shape created in the first step. Figure 3a shows the blunt tip with a very low aspect ratio fabricated at 16 V. The aspect ratio of the probes increased with the rise of the second step etching voltage, and achieved a peak value of about 30 at the second step voltage of 20 V (Figure 3c). Additionally, shape tips with an apex radius of less than 100 nm occurred in this condition (Figure 3c inset). When the voltage increased to 22 V, cylinder-shape probes with more uniform diameter were obtained. The diameter increased to around 1 μm, as well as the tip apex radius (Figure 3d). Further increasing the voltage to 24 V resulted in the increase of the diameter of the probe to 5 μm. Moreover, an arc between the tip and the solution is typically generated by the high voltage at the final etching point, which leads to the melting of the platinum tip and yields a melted ball, as shown in Figure 3e.

Figure 3f shows the platinum probe fabricated at a voltage of 25 V at the first etching step and 20 V at the second step. No electrical discharge etching occurred due to the low voltage used in the first step and, consequently, no “neck-in” structure was formed. The followed second step etching only sharpened the probe tip but could not change the probe shape, resulting in cone-shape probes. This phenomenon indicated that the “neck-in” structure formed by the electrical discharge etching plays a key role in obtaining high-aspect-ratio probes.

**Figure 4:**
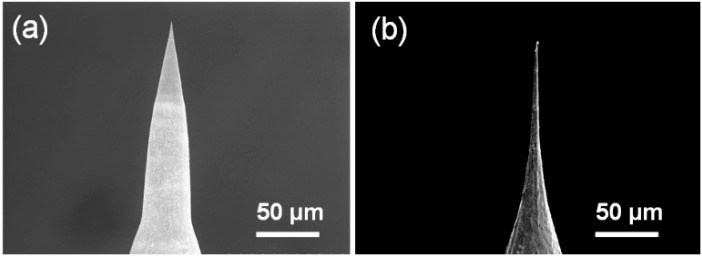
SEM images of the platinum probes fabricated by two-step etching process with different counter-electrode shape. (**a**) Graphite rod electrode; (**b**) Graphite crucible electrode with a diameter of 30 mm.

The shape of the graphite counter-electrode also plays a key role in the aspect ratio of the fabricated probes. Figure 4 shows the platinum probes fabricated with a graphite rod electrode and a 30 mm diameter graphite crucible. When a graphite rod was used, platinum tips with a very low aspect ratio (Figure 4a) were obtained, likely due to the non-uniform electrical field around the platinum wire. However, a long uniform tip (Figure 4b) was fabricated when the graphite crucible was utilized as the counter-electrode. During the etching process, a circular-shaped counter-electrode provided a uniform electrical field around the platinum wire, resulting in a uniform etching on the wire surface and, therefore, a probe with a high aspect ratio.

The relationship between the diameter of the graphite crucible and the yield rate of high-aspect-ratio probes was also investigated to evaluate the instability of the electrochemical discharge etching process. As shown in Table 1, the yield rate of shaped probes with aspect ratios higher than 20 reached 83% when a 30-mm-diameter graphite crucible was used. As the dimension of the graphite crucible decreased to 20 mm, the violent discharging process led to a severe fluctuation of the liquid surface and instability of the fabricated probe shape. Further, increasing the graphite crucible size to 50 mm caused a lower etching current density and longer etching time at the second step, which reduced the length of the fabricated probes.

We also tried to fabricate tungsten tips with this two-stage etching method but were unsuccessful. There is no “neck-in” effect during the first-step etching; however, the tip of the tungsten wire immersed in the solution showed the highest etching rate, indicating a different etching mechanism of tungsten as compared to platinum. Y. Khan *et al.* [21] and S.L. Toh *et al.* [22] fabricated sharp tungsten tips with a dynamic electrochemical etching process with an additional lift-up step and DC [21] or AC [22] power supply. Results from both groups show that a low voltage is necessary to obtain the “neck-in” structure at the liquid-air interface. However, the shape and location of the “neck-in” structure should be controlled by the lift-up step in order to obtain long sharp tungsten tips. It seems that it is hard to obtain high-aspect-ratio tungsten probes by merely adjusting the etching voltages.

## 3. Materials and Methods

The schematic of the experiment setup is shown in Figure 1. The tip material used was a 0.3-mm-diameter platinum wire, cut into 15-mm-long pieces and then ultrasonically cleaned in acetone or alcohol and deionized (DI) water prior to etching. A translation stage was used to control the position of the platinum wire in the electrolyte. Instead of a glass beaker, a high-purity graphite crucible with an electrical resistivity of 13 μΩ·m was used as the container of the electrolyte as well as the counter-electrode for the etching process. The etching electrolyte was a 20 wt. % CaCl_2_ solution. All the solutions were prepared with deionized water.

The tips are formed in a two-step process at two different AC voltages. In order to ensure a quick switch between two voltage values, two variacs were connected in parallel to the platinum wire and the graphite crucible. An MCU-controlled relay was used to switch the two variacs automatically. All experiments were conducted under room temperature. Experiments with graphite rod electrodes were also conducted for comparison. All the experiments were conducted on a vibration insulation stage in order to ensure the stability and consistency of the results. The detailed fabricated process can be found in our previous work [20].

## 4. Conclusions

An electrochemical discharge etching method was investigated for the fabrication of platinum probes with high aspect ratio and nanoscale tip apex radius. Firstly, a “neck-in” structure was fabricated at a voltage of 35 V by the combination effect of electrical discharge etching at the liquid surface and electrochemical etching in the solution. Next, a long sharp probe tip was formed in a gentle etching process at a lower voltage of 18–22 V with graphite crucibles of different sizes used as the counter-electrode. The results indicate that the shape and dimension of the counter-electrode have a significant effect on the aspect ratio and yield rate of the obtained probes. The yield rate of 83% occurred when the 30-mm-diameter graphite crucible was used. Platinum probes with an aspect ratio as high as 30 and a tip apex radius less than 100 nm can occur by using an etching voltage of 20 V and a graphite crucible of 30 mm diameter. The method proposed here provides a simple and rapid technique to fabricate microprobes and needles for micromachining and measurement, micromanipulation and biomedical applications.

## Figures and Tables

**Table 1:** The relationship between the diameter of the graphite crucible and the yield rate of probes with high aspect ratios.

Yield Rate of Probes with Aspect Ratio Higher than 20	Diameter of the Graphite Crucible (mm)	Voltage of the Second Step (V)
40% (4 out of 10)	20	20
83% (25 out of 30)	30	
60% (6 out of 10)	50	
